# Divergence and Model Adequacy, a Semiparametric Case Study

**DOI:** 10.3390/e28070758

**Published:** 2026-07-02

**Authors:** Michel Broniatowski, Justin Moutsouka

**Affiliations:** 1 Laboratoire de Probabilités, Statistique et Modélisation, CNRS, Sorbonne Université, 75005 Paris, France; 2 Sorbonne Université, 75005 Paris, France; moutjust@yahoo.fr

**Keywords:** divergence and model adequacy, semiparametric models, minimum divergence inference, smooth function class

## Abstract

Adequacy for estimation between an inferential method and a model can be defined through two main requirements: firstly the inferential tool should define a well posed problem when applied to the model; secondly the resulting statistical procedure should produce consistent estimators. Conditions which entail these analytical and statistical issues are considered in the context when divergence based inference is applied for smooth semiparametric models under moment restrictions. A discussion is also held on the choice of the divergence, extending the classical parametric inference to the estimation of both parameters of interest and of nuisance.Classical arguments in favor of the omnibus choice of the $L^{2}$ and Kullback Leibler divergences are discussed and motivation for the class of power divergences is presented in the context of the present semi parametric smooth models. A short simulation study illustrates the method.

## 1. Introduction

Classical statistical inference deals with parametric models, which can be seen as finite dimensional manifolds embedded in the class of all probability measures on some measurable space. Therefore, for any value of the parameter, there is a unique distribution in the model; based on a sample governed by an unknown distribution in the model, many of the associated inferential tools (for example, maximum likelihood) have been studied extensively for a considerable amount of models. Obviously the choice of a parametric model results from various sources (theoretical, convenience, rule of thumb, habits, etc.) and often cannot be stated as a truth pertaining to the mechanism which may have generated the data (if such a mechanism exists). Therefore, misspecification has to be considered as well as the resulting properties of the inference under misspecified models; this is a crucial step in the statistical analysis. This question has some overlap with the issues pertaining to robustness, which mainly focus on the role of so-called outliers (or more generally to artifacts in the sampling procedure). Misspecification issues have led to the consideration of tubes around models (in any topologically meaningful sense for the statistical standpoint), with resulting properties of the statistical procedures in this context; note that the inferential procedures keep being fitted to the parametric setting, with no relevance to the neighborhood of the model.

The situation gets even more complex when the model is a collection of subsets of the class of all distributions with non-void interior, and this collection is indexed by a finite dimensional parameter, which is the parameter of interest; a simple example is when each of these subsets consists in all distributions with same expectation, which is the current value of the parameter. This is a special case of the models considered in this paper. Such models are called “semiparametric models”, since a distribution in those is characterized through a finite parameter (of interest), and an infinite dimensional parameter which captures all characteristics of the distribution except the finite dimensional one.

How can statistical criteria handle the complexity of such a context, taking into account the specificity of the infinite dimensional part of the description of the model? Or, phrasing differently, what is a reasonable description of the model (in terms of regularity or other criteria) which still makes inference on the finite dimensional parameter feasible through standard parametric inferential tools, and how should the practical inferential procedure be defined?

We start with some outlook on the minimization of a pseudo-distance between the empirical measure defined by the data set and a model, defined loosely as a collection of probability measures which we consider as candidates for the generic distribution of the data set. This framework is generally referred to as a “divergence based approach”; according to the choice of the divergence (or “pseudo distance”), many classical methods for estimation and testing can be recovered; see [1].

Before entering into our topics in a more detailed way, let us introduce some preliminary definition on the context of this study.

As for the global notation, the space X which bears the data is the Euclidean space $R^{m}$, endowed with its Borel field; all involved probability measures are defined on $\left(X,B\left(X\right)\right)$. In the sequel $M^{1}$ designates the class of all probability measures defined on $\left(X,B\left(X\right)\right)$ and $M^{1}\left(\lambda \right)$, the class of all elements in $M^{1}$ which are absolutely continuous (a.c) with respect to (w.r.t) the Lebesgue measure λ on $R^{m}.$

### 1.1. Semiparametric Models Under Moment Conditions

The models to be considered are defined in two ways.

- Firstly they are defined through constraints on moments; define *l* linearly independent functions
  (1) $$\left(X,\Theta \right)∋\left(x,\theta \right)\to g_{j}\left(x,\theta \right)\;\;1\leq j\leq l$$
  where Θ is included in $R^{d}$ and l≥d.

For any θ let us denote by $M_{\theta }$ the set of all measures in $M^{1}$ defined by

(2) $$M_{\theta }:=\left\{Q\in M^{1}\;\mathrm{such}\;\mathrm{that}\int g_{j}\left(x,\theta \right)dQ\left(x\right)=0,\;1\leq j\leq l\right\}$$

where the integral is performed on the support of *Q*, namely X (or *K* in the sequel). Measures in $M_{\theta }$ therefore satisfy *l* linear constraints. The model M is defined by

(3) $$M=\cup_{\theta \in \Theta }M_{\theta }.$$

We further assume identifiability, meaning that $M_{\theta }\cap M_{\theta^{\prime }}=⌀$ whenever $\theta \neq \theta^{\prime }$.

A simple heteroschedastic example consists in the class of all probability measures *P* defined on R whose expectation θ>0 equals its variance $\int {\left(x-\theta \right)}^{2}dP$; it then holds $g_{1}\left(x,\theta \right)=x-\theta$ and $g_{2}\left(x,\theta \right)={\left(x-\theta \right)}^{2}-\theta$, and hence d=1 and l=2.

- Secondly they are defined through some smoothness condition, which substitutes the usual functional form of parametric inference. Therefore, all distributions in M share some common regularity condition, characterized through regularity properties of their densities with respect to the Lebesgue measure, to be stated in Section 2.2.

Models M satisfying the first set of the above conditions are called “*moment constrained models*”. When, furthermore, the second set of conditions is assumed, we call M a “*smooth moment constrained model*”.

### 1.2. Divergences

A divergence (or discrepancy) between two probability measures *P* and *Q* defined on the same measurable space X equipped with its Borel field $B\left(X\right)$ is a non-negative mapping

$$\left(P,Q\right)\to D\left(Q,P\right)$$

such that $D\left(Q,P\right)=0$ if and only if Q=P. No symmetry is assumed, nor any triangular inequality; therefore, a divergence need not be a distance. Constructions of such functions *D* are numerous; we briefly sketch the present context leading to specific fields of applications in statistics and learning. We refer to [2] for description and further references.

Let us introduce the following definition.

**Definition 1.**  *A divergence D and a moment constrained model M satisfy adequacy when*

*(i) For any distribution $P_{0}$ such that $\inf_{Q\in M}D\left(Q,P_{0}\right)$ is finite, the problem*

$$\arg\underset{Q\in M}{\inf}D\left(Q,P_{0}\right)$$

*is a well posed problem.*

*(ii) Given $P_{n}$ the empirical distribution of an i.i.d. sample under $P_{0}=P_{\theta_{T}}\in M$ the estimator*

$${\hat{\theta }}_{n}:=\arg\underset{\theta \in \Theta }{\inf}\underset{Q\in M_{\theta }}{\inf}D\left(Q,P_{n}\right)$$

*is consistent, namely $\lim_{n\to \infty }{\hat{\theta }}_{n}=\theta_{T}$ in probability.*

Therefore, adequacy holds when conditions on M and on *D* lead to both of the above analytical and statistical properties.

#### Decomposable Divergences

Consider a model $P\subset M^{1}$ defined on X. Say that a divergence

$$\left(P,M^{1}\right)∋\left(Q,P\right)\to D\left(Q,P\right)$$

is decomposable whenever there exist functionals $D^{0}:P\mapsto R$, $D^{1}:M^{1}\mapsto R$ and measurable mappings

(4) $$\rho_{Q}:R^{d}\mapsto R,\;\;\;\;$$

such that for all Q∈P and some $P\in M^{1}$ the expectation $\int \rho_{Q}dP$ exists and

(5) $$D\left(Q,P\right)=D^{0}\left(Q\right)+D^{1}\left(P\right)+\int \rho_{Q}dP.$$

It is customary to restrict *P* to the subset of $M^{1}$ for which the expectation $\int \rho_{Q}dP$ exists for all *Q* in P. Examples of decomposable divergences are numerous. Those include both the L^2^ and the Kullback–Leibler divergences, but general Csiszar Ali Silvey Morimoto divergences (CASM) (or so-called *f*-divergences) are not captured through this definition; we refer to [3] and to [4] for definitions, examples and properties; associated estimators are defined as minimizers of $D(Q,P_{n})$ upon *Q*, where $P_{n}$ designates the empirical distribution pertaining to the observed data set $\left(X_{1},\ldots ,X_{n}\right).$ Looking at (5) we see that decomposable divergences lead to simple M-estimators of *Q* through substitution of *Q* by $P_{n}$, with

$$Q_{n}:=\arg\min_{Q\in P}D^{0}\left(Q\right)+\frac{1}{n}\sum_{i=1}^{n}\rho_{Q}\left(X_{i}\right)$$

whenever defined.

**Remark 1.**  *When Q runs in a parametric family $P:=\left\{P_{\theta },\theta \in \Theta \right\}$ then the function $\theta \to \rho_{\theta }$ is reminiscent of the monotone embedding formalism for generalized CASM or Bregman divergences; see [5] and references therein, and corresponding formalism in generalized exponential families under moment constraints in [6,7] among others.*

### 1.3. On the Choice of the Divergence

#### 1.3.1. The Need for a Specific Approach

The identification of pertinent distances for inference in models defined through moment conditions has been considered by Csiszar [8]; the general setting is that when all distributions involved share the same finite support *K* and when Θ is restricted to a single value, the resulting ill posed inverse problem is somehow similar to the inferential problem stated in the empirical likelihood paradigm with given moment condition (hence for *moment constrained models*); see the next paragraph for definitions. The work in [8] considers projection rules defined through minimization of a pseudo-distance between a given distribution (the empirical distribution in the statistical context) and the set of all probability vectors satisfying the moment constraint. Basic assumptions which should be fulfilled by the admissible rules include the so-called “locality property”; in relation to the present article, it states that splitting X into two disjoint subsets $K_{1}$ and $K_{2}$ such that $X=K_{1}\cup K_{2}$, the corresponding solutions of those moment problems restricted to $K_{1}$ and $K_{2}$ with same constraint can be assembled through mixing to produce the solution of the initial moment problem on X. Csiszar [8] identifies all projection rules as pseudo-distance minimization operators which satisfy the locality property with some further natural axioms; those rules are restricted to the $L^{2}$ projection operator, or to the Kullback–Leibler operator (which yields the EL paradigm); although developed only for finitely supported models, these arguments carry over to the continuous case. In the semiparametric case considered here (namely the *smooth moment constrained model*), the locality property cannot be considered as a necessary criterion for the definition of the projection rule. Indeed, the global regularity constraint on the density of the solution to the moment problem cannot generally be recovered through local ones: for example, assuming Lipschitz regularity of the densities of elements in M on $K_{1}$ and $K_{2}$ does not yield Lipschitz regularity on X. Henceforth, we are left with the choice of the projection rule, and the quest for the incidence of its properties on the solution of the moment problem under regularity assumptions remains open; this motivates this paper.

#### 1.3.2. Alternative Procedures

As mentioned earlier the question which we consider is the following: starting with a discrepancy measure, what are the admissible models (in the range of smooth moment constrained ones) for which optimization of the given discrepancy is a valid procedure? For sake of completeness, we shortly indicate some plausible alternative techniques, with indications about their limitations.

The inference on θ in models defined by moment conditions can be performed in a natural way for a number of statistical criteria. Indeed, for example, for Cressie Read criteria, or more generally for CASM-type criteria, a simple plug in of the empirical measure $P_{n}$ in place of *P* in the divergence D(Q,P) allows us to minimize it on $M_{\theta }$ for a given θ, and then to optimize upon θ. This is due to the fact that the minimizer of $D(Q,P_{n})$ on $M_{\theta }$ has support included in the sample points $\left\{X_{1},\ldots ,X_{n}\right\}$. Therefore, the seemingly formidable search for this minimization problem boils down to a finite dimensional one, on the simplex of $R^{n}.$ Such is the core argument for Empirical Likelihood (EL) methods and their extensions (see, e.g., [9] or [10] for a general CASM approach). All minimum empirical divergence methods (therefore including EL) aim at assessing whether the model M is valid and at the estimation of $\theta_{T}$, the value of the parameter whenever $P_{0}$, which designates the distribution of the data, equals $P_{\theta_{T}}$. So they do not provide any knowledge on the density of $P_{\theta_{T}\;}$ (whenever $P_{0}=P_{\theta_{T}}$ belongs to M) nor on the density of the projection of $P_{0}$ on M, taking into account the very definition of the model. Some penalized version of EL has been proposed (see, e.g., [11] and references therein) but the context therein seems somehow different from ours. Extending the parametric setting to a smoothed semiparametric one, it is possible to make inference both on $\theta_{T}$ and on the density of $P_{\theta_{T}}$. We therefore take advantage of the very nature of the chosen criterion to circumvent the obstacle due to the assumed regularity of the distribution of the data. The same type of approach could be adopted making use of the minimization of Bregman divergences or others.

Obviously from an operational standpoint one could suggest making use of the method of EL as a first step, hence making use of the Kullback–Leibler projection rule, leading to a distribution $Q_{{\hat{\theta }}_{n}\;}$ supported by the sample $\left(X_{1},\ldots ,X_{n}\right)$ with ${\hat{\theta }}_{n}$ converging to $\theta_{T}$ as *n* tends to infinity, and then projecting $Q_{{\hat{\theta }}_{n}\;}$ on the class of distributions with density (w.r.t the Lebesgue measure) satisfying the prescribed smoothness requirement. However, this latest projection might lead to the loss of the moment constraint; furthermore, it bears a number of major difficulties. These include the choice of a projection rule, which would handle the absolute continuity obstacle, typically by making use of some smoothing technique. There exists a huge literature on smoothed estimation in non-parametric or semiparametric models through penalization techniques, which is outside the scope of this paper.

### 1.4. A Class of Adequate Divergences, the Power Divergences

Because of the assumed regularity of the densities of measures in M we consider divergences D(Q,P) which are explicit functionals of densities, excluding therefore CASM divergences (except the Kullback–Leibler one) for which the density q:=dQ/dλ appears only through its ratio with the density of P. We turn therefore to the Bregman class, and consider the subclass of power divergences $D_{\alpha }$ introduced by Basu Hjort, Harris and Jones (BHHJ) [12], and which has been embedded in a flexible family of divergences by Chicoki and Amari [13]; see also [14] for robust Bregman divergences extending the BHHJ class, and [15] for a comprehensive approach with applications. We will stick to the basic form $D_{\alpha }$, which proves to be a pertinent candidate for inference in parametric models. It also bears the benefit of being indexed by a single parameter α, which can be confronted with the smoothness of the model. Also, the power divergence $D_{\alpha }$ is decomposable, which allows for simple application of classical results on M-estimators obtained by plug in.

We briefly recall the main features of $D_{\alpha }$, which is defined by

$$D_{\alpha }\left(Q,P\right):=\int \varphi \left(q\left(x\right),p\left(x\right)\right)dx$$

where

$$\varphi \left(u,v\right)=\frac{1}{\alpha }u^{\alpha +1}-\left(1+\frac{1}{\alpha }\right)u^{\alpha }\times v+v^{\alpha +1}.$$

Note that in accordance with some widely accepted notation in Information theory, we denote $Q\in \Omega \to D_{\alpha }\left(Q,P\right)$ the projection rule which maps the fixed measure *P* in $M^{1}\left(\lambda \right)$ over some subset Ω of $M^{1}\left(\lambda \right)$. This differs from the original notation in [12], where the notation is reversed and the mapping is denoted $Q\to D_{\alpha }\left(P,Q\right)$. The same notational ambiguity is unfortunately common in the global literature on divergences, and leads to some confusion, for example between CASM divergences and their conjugates (Neyman and Pearson Chi square, Kullback–Leibler and Likelihood divergences, etc.).

Indeed, the BHHJ divergence is decomposable. In the semi- or non-parametric context, it is more advisable to make use of a generic notation, namely

$$D^{0}\left(Q\right):=\int q^{\alpha +1}d\lambda$$

$$D^{1}\left(P\right):=\frac{1}{\alpha }\int p^{\alpha +1}d\lambda$$

$$\rho_{q}:=-\left(1+\frac{1}{\alpha }\right)q^{\alpha }.$$

from which (5) holds.

Minimization on *Q* over some class $M_{\theta }$ included in $M^{1}\left(\lambda \right)$ is equivalent to the minimization of the criterion

(6) $$R_{\alpha }\left(Q,P\right)=D^{0}\left(Q\right)+\int \rho_{q}dP$$

over $M_{\theta },$ which allows for the plug in of $P_{n}$ in place of *P*, resulting in the common M-estimator framework.

We refer to [3,4] for definition, properties and extensions. We will consider values of α in $\left(0,1\right]$, which ensures that for all nonnegative *v* the mapping u→φ(u,v) defined on $R^{+}$ is strictly convex; the case when α=0 is the Kullback–Leibler case, not considered here; the case when α=1 is the L^2^ case, which is accessible through our approach.

The developed form of $D_{\alpha }\left(Q,P\right)$ is therefore

(7) $$D_{\alpha }\left(Q,P\right)=\int \left\{q^{\alpha +1}\left(v\right)-\left(1+\frac{1}{\alpha }\right)q^{\alpha }\left(v\right)p\left(v\right)+\frac{1}{\alpha }p^{\alpha +1}\left(v\right)\right\}dv.\;\;$$

The rationale for the BHHJ class in parametric inference in a model $P:=\left\{P_{\theta }\in M^{1}\left(\lambda \right),\theta \in \Theta \right\}$ lies in the fact that whenever the integral in the above display does not depend on the parameter θ, as holds for location models, then minimizing upon θ in $R_{\alpha }\left(P_{\theta },P_{n}\right)$ amounts to smoothing the usual likelihood score by a factor $p_{\theta }^{\alpha -1}$, which damps the role of outliers in the estimating equation.

This procedure has been developed extensively and leads to classical limit results for estimation and testing in parametric contexts; see Theorem 2 in [12]. The performance of this approach has been compared to similar treatments making use of CASM divergences, both under the model and under misspecification; globally speaking, performances of either CASM divergence approach or power divergence approach are quite similar (same limit distribution of the estimator and of the test statistics as for the maximum likelihood approach (which falls in the field of CASM divergences but not in the field of power ones for α in $\left(0,1\right]$), nearly similar results in simulation runs on small or medium size samples). Comparing properties between BHHJ divergences with various values of α and corresponding ones for the power divergences of Cressie-Read type with various parameters γ (which describe the most commonly used subclass in the CASM divergences) allows one to obtain reasonably robust estimators under contamination, as measures through the Influence function; see [4]. These performances make them good candidates for inferential tools in the semiparametric framework.

#### Smooth Semiparametric Models Under Moment Conditions, Specificity of the Present Approach

Our standpoint is to propose a procedure which by its very nature produces a smooth density which satisfies the model assumption. The drawback clearly lies in appropriate algorithms taking into account the complexity of the required regularity of the model. A short simulation at the end of the paper illustrates the behavior of the estimator in a very simple case; however, the present paper provides the necessary setup which has to be developed, and which results as a common frame for similar proposals under a similar semiparametric framework. For example, we may consider classes of unimodal densities with unknown mode, or models with densities defined by conditions on their L-moments [16,17]; in all those examples, the functional context is similar to the one considered here. The class of densities embedded in a function class (denoted *E* hereunder) has to be tailored accordingly.

Consider the estimation of θ in the context of the smooth moment constrained model M; this yields to a two-step minimization. The first one consists in the search for the minimizer $Q_{\theta }$ of $R_{\alpha }\left(Q,P_{n}\right)$ for *Q* in the smooth subset of $M_{\theta }$, and the subsequent minimization should select the value of θ which solves $\min_{\theta }$$R_{\alpha }\left(Q_{\theta },P_{n}\right)$ where $Q_{\theta }$ solves the first minimization, whenever possible. Firstly the model should be such that all minimization procedures should be well defined; additional regularity assumptions on the model, with respect to the variation of θ in Θ, will be necessary in order to perform the second optimization. The hypotheses in this paper are not meant to meet the highest generality, but merely to address a simple framework where adequacy can be considered and discussed.

The problem at hand can therefore be written as

(8) $${\hat{\theta }}_{n}:=\arg\min_{\theta \in \Theta }\min_{Q\in M_{\theta }}R_{\alpha }\left(Q,P_{n}\right),$$

where for all θ, $M_{\theta }$ consists in a family of distributions with densities w.r.t the Lebesgue measure, with some prescribed regularity. We need to introduce some description on the model with adequate notation; this is done in the next Section.

## 2. Notation and Properties of the Smooth Semiparametric Model

### 2.1. Constraints

All distributions in this model are defined on a compact subset *K* of $R^{m}.$ The linearly independent functions $\left(g_{1},\ldots ,g_{l}\right)$ introduced in (1) should satisfy some basic requirements. Each of the functions $g_{i}$ is defined on *K* with values in R.; hence, $g:={\left(g_{1},\ldots ,g_{l}\right)}^{T}$ is defined on K×Θ with values in $R^{l}.$ The parameter space Θ is a compact subset in $R^{d}$.

We assume that for all θ the mapping

(9) $$\left(x,\theta \right)⟶g\left(x,\theta \right)\;\;\;\mathrm{is}\;\mathrm{continuous}\;\mathrm{on}\;\;\;int\left(K\right)\times int\left(\Theta \right).$$

It follows that all functions $g_{l}$’s are uniformly bounded

(10) $$\underset{\theta }{\sup}\underset{x\in K}{\sup}∥g(x,\theta )∥<\infty$$

where $∥x∥$ designates the usual norm in $R^{l}.$

It also follows from (9) that uniform continuity of *g* holds in the sense that as $\theta_{n}\to \underline{\theta }$

$$\lim_{n\to \infty }\underset{x\in K}{\sup}∥g\left(x,\theta_{n}\right)-g\left(x,\underline{\theta }\right)∥=0.$$

### 2.2. Regularity and Smoothness Assumptions

The semiparametric model $M_{E}$ will be assumed to consist in regular measures, in the sense that they should have density with respect to the Lebesgue measure λ on *K*, and that their densities should be smooth. This is formalized as follows.

Let $M_{K}^{1}$ be the class of all probability measures with support *K*, and $M_{K}^{1}\left(\lambda \right)$ the class of all probability measures in $M_{K}^{1}$ which are a.c. w.r.t λ.

Denote $C_{b}\left(K\right)$, the class of all continuous bounded functions defined on *K*. We now define a subset *E* of $C_{b}\left(K\right)$, endowed with the metric induced by the sup norm on *K*; for *q* and $q^{\prime }$ in *E*, denote

$$d\left(q,q^{\prime }\right):=\underset{x\in K}{\sup}\left|q\left(x\right)-q^{\prime }\left(x\right)\right|.$$

Two conditions will be assumed on *E*.

1-We assume that *E* is uniformly bounded on *K*, namely

(11) $$\underset{q\in E}{\sup}\underset{x\in K}{\sup}\left|q(x)\right|<+\infty .$$

2-We denote by (12) the following condition, which allows for standard application of limit results for classes of functions in order to prove consistency of the present estimation procedure.

(12) $$\underset{q\in E}{\sup}\underset{x,y\in K}{\sup}\frac{\left|q^{\alpha }\left(x\right)-q^{\alpha }\left(y\right)\right|}{\left|x-y\right|}\leq M$$

(i) When the class *E* is lower bounded on *K* by some positive γ, i.e., when

$$\underset{q\in E}{\inf}\underset{x\in K}{\inf}q\left(x\right)>\gamma >0$$

then we assume that $q^{\delta }$ is Lipschitz uniformly over *E* for some $\delta \in \left(0,1\right]$; this implies that $q^{\delta }$ is Lipschitz for all δ in $\left(0,1\right]$ and hence for α.

(ii) If the class *E* cannot be uniformly lower bounded on *K* then we assume that $q^{\delta }\;$ is Lipschitz uniformly on *E* for some δ:0<δ<α.

Under (12)(i) (12) clearly holds, as under (12)(ii) since then $\sup_{p\in E}\sup_{x\in K}\left|{\left(p^{\alpha }\right)}^{\prime }\left(x\right)\right|$ is bounded.

**Remark 2.**  *Condition (12) implies that the class E is equicontinuous: for all ε>0, there exists δ>0 such that for all q in E,*

$$\underset{|x-x^{\prime }|<\delta }{\sup}\left|q\left(x\right)-q\left(x^{\prime }\right)\right|<\varepsilon .$$

Define E the class of all non-negative finite measures *Q* on *K* with density q:=dQ/dλ in E.

For each θ consider the submodel

$$M_{\theta }:=\left\{Q\in M_{K}^{1}\;\mathrm{such}\;\mathrm{that}\int g\left(x,\theta \right)dQ\left(x\right)=0\right\},$$

and its smooth counterpart

$$M_{\theta_{E}}:=M_{\theta }\cap E,$$

which we assume to be non-void. We define the model M through

$$M=\cup_{\theta \in \Theta }M_{\theta }$$

and the smooth version of M is defined by

$$M_{E}=\cup_{\theta \in \Theta }M_{\theta }\cap E=\cup_{\theta }M_{\theta_{E}}$$

which we call the smooth moment constrained model.

As quoted before the first additional condition is an identifiability property of the model M with respect to θ:

For $\theta \neq \theta^{\prime }$,

(13) $$M_{\theta }\cap M_{\theta^{\prime }}=\emptyset .$$

We now state that for any θ all smooth densities in $M_{\theta }$ can be distinguished from their counterparts in $M_{\theta^{\prime }}$ when $\theta^{\prime }\neq \theta .$ This can be phrased as follows: the collection of smooth submodels $M_{\theta_{E}}$ is well separated in the sense that for any positive ϵ there exists some positive δ such that

(14) $$\left(d(\theta ,\theta^{\prime })>\epsilon \right)\;\;\mathrm{implies}\;\;\left(\underset{\left\{Q\in M_{\theta_{E}},Q^{\prime }\in M_{\theta_{E}^{\prime }}\right\}}{\inf}d\left(q,q^{\prime }\right)>\delta \right).$$

where we have denoted q:=dQ/dλ and $q^{\prime }:=dQ^{\prime }/d\lambda .$ The same notation will be used in the sequel: for example, for $Q_{n}$ in $M_{E}$, $q_{n}$ will designate $dQ_{n}/d\lambda$, etc.

**Example 1.**  *g(x)=x−θ,$M_{\theta }=\left\{Q:\int_{K}xdQ\left(x\right)=\theta \right\}$ and clearly $M_{\theta }\cap M_{\theta }^{\prime }=\emptyset .$ Whenever $\left|\int_{K}x\left(q\left(x\right)-q^{\prime }\left(x\right)\right)dx\right|>\varepsilon$ then $\int_{K}\left|q\left(x\right)-q^{\prime }\left(x\right)\right|dx>\varepsilon /\left|K\right|$, where $\left|K\right|$ denotes the volume of K, and therefore $d(q,q^{\prime })>\delta$ for some δ>0.*

### 2.3. The Estimator

Given an i.i.d. sample $\left(X_{1},X_{2},\ldots ,X_{n}\right)$ such that $X_{1}$ has distribution $P_{\theta_{0}}$$\in M_{\theta_{0}\;}$ for some $\theta_{0}\in \Theta$, we intend to provide an estimator for $\theta_{0}$ minimizing the pseudo-distance between $P_{n}$ and $M_{E}$ where

$$P_{n}:=\frac{1}{n}\sum_{i=1}^{n}\delta_{X_{i}}$$

is the empirical measure pertaining to the i.i.d. sample $\left(X_{1},X_{2},\ldots ,X_{n}\right)$. Note that the estimation is performed in the smooth model $M_{E}$ and not in M.

We introduce the estimator of $\theta_{0}$ in $M_{E}$ by

(15) $${\hat{\theta }}_{n}:=\arg\underset{\theta }{\inf}\underset{Q\in M_{\theta_{E}}}{\inf}D_{\alpha }\left(Q,P_{n}\right).$$

Formula (15) provides a natural estimate of $\theta_{0}$ if $P_{\theta_{0}}\in M_{\theta_{0_{E}}}$. Indeed, under the identifiability conditions (13) and (14) we prove that the above estimator converges to $\theta_{0}=\arg\inf_{\theta }\inf_{Q\in M_{\theta }}D_{\alpha }\left(Q,P_{\theta_{0}}\right)$ (see Theorem 1 and Theorem 5).

In the alternative case that $P_{\theta_{0}}\in M_{\theta_{0}}$ but $P_{\theta_{0}}\notin E$, then Formula (15) defines an estimator of some $\tilde{\theta }:=\arg\inf_{\theta }\inf_{Q\in M_{\theta_{E}}}D_{\alpha }\left(Q,P_{\theta_{0}}\right)$. Hence, $P_{\tilde{\theta }}$ is the $D_{\alpha }$-projection of $P_{\theta_{0}}$ on $M_{E}$, and $\tilde{\theta }$ may be different from $\theta_{0}$ but still $P_{\tilde{\theta }}$ represents a proxy of $P_{\theta_{0}}$ in the smooth model. We will consider a natural condition which entails that $\tilde{\theta }=\theta_{0}$ (see Theorem 1).

### 2.4. Adequacy

For $D_{\alpha }$ and $M_{E}$ adequacy, since (5) holds and making use of $R_{\alpha }$ defined in (6), Definition 1 takes the following form:

**Definition 2.**  *The power divergence $D_{\alpha }$ and the smooth moment constrained model $M_{E}$ satisfy adequacy when:*

*(i) For any distribution $P_{0}$ such that $\inf_{Q\in M_{E}}D_{\alpha }\left(Q,P_{0}\right)$ is finite, the problem*

$$\arg\underset{Q\in M_{E}}{\inf}D_{\alpha }\left(Q,P_{0}\right)=\arg\underset{Q\in M_{E}}{\inf}R_{\alpha }\left(Q,P_{0}\right)$$

*is a well posed problem.*

*(ii) Given $P_{n}$ the empirical distribution of an i.i.d. sample under $P_{0}=P_{\theta_{T}}\in M_{E}$, the estimator*

$${\hat{\theta }}_{n}:=\arg\min_{\theta \in \Theta }\min_{Q\in M_{\theta_{E}}}R_{\alpha }\left(Q,P_{n}\right)$$

*is consistent in probability, and $\lim_{n\to \infty }{\hat{\theta }}_{n}=\theta_{T}$.*

## 3. Projection and Regularization

We denote $P_{0}$ the distribution of the variable $X_{1}$. In this section we consider both cases $P_{0}\in M_{\theta_{0}}$ and $P_{0}\in M_{\theta_{0_{E}}}$ for some $\theta_{0}$.

Suppose that the following condition holds:

(16) $$\underset{Q\in M_{\theta_{0_{E}}}}{\inf}D_{\alpha }\left(Q,P_{0}\right)<\underset{Q\in M_{\theta_{E}}}{\inf}D_{\alpha }\left(Q,P_{0}\right)$$

for all $\theta \neq \theta_{0}$, whenever $P_{0}$ belongs to $M_{\theta_{0}}$ which formalizes the fact that $P_{0}$ is approximated smoothly with a better score in $M_{\theta_{0_{E}}}$ than in any $M_{\theta_{E}}$, whenever $P_{0}$ belongs to $M_{\theta_{0}}.$ Condition (16) connects the smoothness condition of the model with the divergence criterion. It implies that projecting $P_{0}$ on M or on $M_{E}$ identifies $\theta_{0}$ in a unique way, as stated in the following result, to be proved in Appendix A.

**Theorem 1.**  *Under (16) it holds, whenever $P_{0}$ belongs to M or to $M_{E},$*

(17) $$\theta_{0}=\arg\underset{\theta }{\inf}\underset{Q\in M_{\theta_{E}}}{\inf}D_{\alpha }\left(Q,P_{0}\right)=\arg\underset{\theta }{\inf}\underset{Q\in M_{\theta }}{\inf}D_{\alpha }\left(Q,P_{0}\right).$$

Before handling inference we need to explore some properties of minimum pseudo-distance approximations in $M_{E}.$ We will make use of a number of definitions, which we quote now. For fixed *P* in $M_{E}$, the divergence $D_{\alpha }{\left(.,P\right)|}_{E}$ is the restriction of $Q\to D_{\alpha }\left(Q,P\right)$ on $M_{E}$.

For fixed θ, let therefore the projection of *P* on $M_{\theta_{E}}$ be

$$Q_{\theta }^{*}=\arg\underset{Q\in M_{\theta_{E}}}{\inf}D_{\alpha }{\left(Q,P\right)|}_{E}$$

whenever defined.

Since for $Q\in M_{E}$

$$D_{\alpha }{\left(Q,P\right)|}_{E}=D_{\alpha }\left(Q,P\right)$$

it holds

$$\arg\underset{Q\in M_{\theta_{E}}}{\inf}D_{\alpha }\left(Q,P\right)=\arg\underset{Q\in M_{\theta_{E}}}{\inf}D_{\alpha }{\left(Q,P\right)|}_{E}=Q_{\theta }^{*}.$$

We first set some general definitions.

**Definition 3.**  *Let *Ω* be some subset of $M^{1}$. The α-divergence between the set *Ω* and a p.m. P is defined by*

$$D_{\alpha }\left(\Omega ,P\right):=\underset{Q\in \Omega }{\inf}D_{\alpha }\left(Q,P\right).$$

A probability measure $Q^{*}\in \Omega$, such that $D_{\alpha }\left(Q^{*},P\right)<\infty$ and

$$D_{\alpha }\left(Q^{*},P\right)\leq D_{\alpha }\left(Q,P\right)\;\;\mathrm{for}\;\mathrm{all}\;\;Q\in \Omega$$

*is called a projection of P on *Ω*. This projection may not exist, or may not be defined uniquely.*

**Definition 4.**  *The sequence of functions $q_{n}\in E$ tends to q strongly if and only if*

$$\underset{x\in K}{\sup}\left|q_{n}\left(x\right)-q\left(x\right)\right|\to 0.$$

Let ${\left(Q_{n}\right)}_{n}\subset M_{E}$; if there exists some *q* in *E* such that

(18) $$\underset{x\in K}{\sup}\left|q_{n}\left(x\right)-q\left(x\right)\right|\to 0,$$

then we say that $Q_{n}$ converges strongly to a non-negative finite measure *Q* such that $Q\left(A\right)=\int 1_{A}\left(x\right)q\left(x\right)dx$ for all $A\in B(R^{m})$. Denote $\left(Q_{n}\underset{st}{⟶}Q\right)$ when (18) holds.

## 4. Projection: Existence and Uniqueness

Let *P* belong to $M_{K}^{1}\left(\lambda \right)$ such that $\inf_{Q\in M}$$D_{\alpha }\left(Q,P\right)$ is finite.

We need some preliminary result pertaining to the properties of $M_{E}.$

### 4.1. Closure of $M_{E}$

Conditions (11) and (12) imply that due to the Arzela-Ascoli Theorem, the set *E* is pre-compact when endowed by the strong topology (see Definition 3).

Let $\left(Q_{n}\right)$ be a family of probability measures on *K*; it holds

**Proposition 1.**  *$M_{E}$ is relatively compact in E endowed with the strong topology.*

Let $\left\{n_{j}\right\}$$\subset \left\{n\right\}$ and $\frac{dQ_{n_{j}}}{d\lambda }\left(x\right)=q_{n_{j}}\left(x\right)$, and $\sup_{x\in K}\left|q_{n_{j}}\left(x\right)-q\left(x\right)\right|⟶0$ then $\left(Q_{n_{j}}\right)$ converges to some p.m *Q* and $Q\left(A\right)=\int_{A}q\left(x\right)d\lambda \left(x\right)$ for all *A* in B(K).

Indeed,

$$\begin{array}{ccc} \left|Q_{n_{j}}\left(A\right)-\int_{A}q\left(x\right)d\lambda \left(x\right)\right| & = & \left|\int 1_{A}\left(x\right)q_{n_{j}}\left(x\right)d\lambda \left(x\right)-\int 1_{A}\left(x\right)q\left(x\right)d\lambda \left(x\right)\right|\\ & \leq & \underset{x\in K}{\sup}\left|q_{n_{j}}\left(x\right)-q\left(x\right)\right|\lambda \left(A\right)⟶0. \end{array}$$

So ${\left(Q_{n_{j}}\right)}_{j\geq 1}$ converges to *Q*, such that $q\left(x\right)=\frac{dQ}{d\lambda }\left(x\right)$. That *Q* is a probability measure is a consequence of the Prohorov Theorem (see, e.g., [18]) since ${\left(Q_{n}\right)}_{n\geq 1}$ is a tight family of p.m’s.

It follows that

**Theorem 2.**  *Under (*9*),(*10*) the set $M_{E}$ is closed for the strong topology of convergence stated in Definition 3.*

The proof of Theorem 2 is in Appendix A.

### 4.2. Existence and Uniqueness of the $D_{\alpha }$-Projection of *P* on $M_{E}$

Assume *P* in $M_{K}^{1}\left(\lambda \right).$ Let a>0 and

$$A_{E}\left(a\right):=\left\{Q\in M_{E}:D_{\alpha }\left(Q,P\right)\leq a\right\}$$

be the a-level set of the divergence $Q\to D_{\alpha }\left(Q,P\right).$

It holds

**Proposition 2.**  *For any $\alpha \in \left(0,1\right]$ the divergence function $Q\mapsto D_{\alpha }\left(Q,P\right)$ from $M_{K}^{1}\left(\lambda \right)$ to [0,+∞] is l.s.c for the strong topology.*

The proof of the above proposition is in Appendix A.

It also holds (see the proof in Appendix A).

**Proposition 3.**  *For all a>0, the level set $A_{E}\left(a\right)$ of $Q\to D_{\alpha }\left(Q,P\right)$ is compact in the strong topology. Furthermore, for any θ in *Θ*,*

$$Q^{*}=\arg\underset{Q\in M_{\theta_{E}}}{\inf}D_{\alpha }\left(Q,P\right)$$

*exists and is unique.*

Consider now the $D_{\alpha }$-projection of $P\in M_{K}^{1}\left(\lambda \right)$ on a convex subset Ω in $M_{E}.$ Making use of Propositions 2 and 3, the following proposition holds.

**Theorem 3.**  *For any closed convex set *Ω* in $M_{E}$ the $D_{\alpha }$ projection of P on *Ω* exists and is unique.*

**Proof.**  Indeed, let

$$a:=\underset{Q\in \Omega }{\inf}D_{\alpha }\left(Q,P\right)$$

and ε>0. Then $A_{E}\left(a+\varepsilon \right)\cap \Omega \neq \emptyset$. Since Ω is closed and $A_{E}\left(a+\varepsilon \right)$ is compact, the existence of the projection follows.

Uniqueness is due to strict convexity. □

## 5. Minimum Pseudo-Distance Estimator

We now provide a formal definition of the minimum power distance estimator in the smooth moment constraint model, from which asymptotic properties will be derived.

Let $X_{1},\ldots ,X_{n}$ denote an i.i.d. sample of a random vector $X\in R^{m}$ with distribution $P_{0}$ in $M_{K}^{1}\left(\lambda \right)$. Let $P_{n}\left(.\right)$ be the empirical measure pertaining to this sample, namely

$$P_{n}\left(.\right):=\frac{1}{n}\sum_{i=1}^{n}\delta_{X_{i}}\left(.\right)$$

where $\delta_{x}\left(.\right)$ denotes the Dirac measure at point *x*. We define

$$\begin{array}{cc} D_{\alpha }\left(M_{\theta_{E}},P_{0}\right) & =\underset{Q\in M_{\theta_{E}}}{\inf}D_{\alpha }\left(Q,P_{0}\right)\\ \; & =\underset{Q\in M_{\theta_{E}}}{\inf}\left\{\int \left(q^{\alpha +1}\left(x\right)-\left(1+\frac{1}{\alpha }\right)q^{\alpha }\left(x\right)p_{0}\left(x\right)+\frac{1}{\alpha }p_{0}^{\alpha +1}\left(x\right)\right)dx\right\}. \end{array}$$

Since optimization only pertains to *Q* define in the following

$$\begin{array}{cc} R_{\alpha }\left(M_{\theta_{E}},P_{0}\right) & :=\underset{Q\in M_{\theta_{E}}}{\inf}R_{\alpha }\left(Q,P_{0}\right)\\ \; & =\underset{Q\in M_{\theta_{E}}}{\inf}\left\{\int \left(q^{\alpha +1}\left(x\right)-\left(1+\frac{1}{\alpha }\right)q^{\alpha }\left(x\right)p_{0}\left(x\right)\right)dx\right\}, \end{array}$$

and the “plug-in” estimate of $R_{\alpha }\left(M_{\theta_{E}},P_{0}\right)$ through

$$\begin{array}{cc} {\hat{R}}_{\alpha }\left(M_{\theta_{E}},P_{0}\right) & :=\underset{Q\in M_{\theta_{E}}}{\inf}R_{\alpha }\left(Q,P_{n}\right)\\ \; & =\underset{Q\in M_{\theta_{E}}}{\inf}\left\{\int q^{\alpha +1}\left(x\right)dx-\left(1+\frac{1}{\alpha }\right)\int q^{\alpha }\left(x\right)dP_{n}\left(x\right)\right\}\\ \; & =\underset{Q\in M_{\theta_{E}}}{\inf}\left\{\int q^{\alpha +1}\left(x\right)dx-\left(1+\frac{1}{\alpha }\right)\frac{1}{n}\sum_{i=1}^{n}q^{\alpha }\left(X_{i}\right)\right\} \end{array}$$

In the same way,

$$\begin{array}{cc} R_{\alpha }\left(M,P_{0}\right) & :=\underset{\theta \in \Theta }{\inf}\underset{Q\in M_{\theta_{E}}}{\inf}R_{\alpha }\left(Q,P_{0}\right)\\ \; & =\underset{\theta \in \Theta }{\inf}\underset{Q\in M_{\theta_{E}}}{\inf}\left\{\int q^{\alpha +1}\left(x\right)dx-\left(1+\frac{1}{\alpha }\right)\int q^{\alpha }\left(x\right)dP_{0}\left(x\right)\right\} \end{array}$$

can be estimated by

$${\hat{R}}_{\alpha }\left(M,P_{0}\right):=\underset{\theta \in \Theta }{\inf}\underset{Q\in M_{\theta_{E}}}{\inf}\left\{\int q^{\alpha +1}\left(x\right)dx-\left(1+\frac{1}{\alpha }\right)\frac{1}{n}\sum_{i=1}^{n}q^{\alpha }\left(X_{i}\right)\right\}$$

Since

$$\arg\underset{Q\in M_{\theta_{E}}}{\inf}D_{\alpha }\left(Q,P_{0}\right)=\arg\underset{Q\in M_{\theta_{E}}}{\inf}R_{\alpha }\left(Q,P_{0}\right)$$

for any θ

$$\arg\underset{Q\in M_{\theta_{E}}}{\inf}R_{\alpha }\left(Q,P_{0}\right)$$

exists and is unique (whether $P_{0}\in \cup M_{\theta_{E}}$ or not).

We will consider estimators of $\theta_{0}$ where $P_{0}=P_{\theta_{0}}$ for some $\theta_{0}\in \Theta$; this corresponds to the fact that $P_{0}\in M$. In this case by uniqueness of $\arg\inf_{\theta \in \Theta }R_{\alpha }\left(M_{\theta_{E}},P_{0}\right)$ and since the infimum is reached at $\theta =\theta_{0}$ under the model, $\theta_{0}$ is estimated through

$${\hat{\theta }}_{n}:=\arg\underset{\theta \in \Theta }{\inf}\underset{Q\in M_{\theta_{E}}}{\inf}\left\{\int q^{\alpha +1}\left(x\right)dx-\left(1+\frac{1}{\alpha }\right)\frac{1}{n}\sum_{i=1}^{n}q^{\alpha }\left(X_{i}\right)\right\}.$$

## 6. Asymptotic Properties

### 6.1. Consistency

The pseudodistances $D_{\alpha }$ will be applied in the standard statistical estimation model with i.i.d observations $X_{1},\ldots ,X_{n}$ (with empirical measure $P_{n}$) governed by $P_{0}$, which is assumed to belong to $M_{E}$. In this case $P_{0}=P_{\theta_{0}}$ for some $\theta_{0}$ in Θ (see Remark 3 hereunder). The present inference amounts to produce both an estimate of the finite dimensional parameter $\theta_{0}$ and the density $p_{\theta_{0}}$, which is assumed to belong to *E*. For any θ a first candidate $Q_{n}\left(\theta \right)$ with smooth density in *E* is obtained as the minimum divergence projection of $P_{n}$ on the smooth submodel $M_{\theta_{E}}$ (Inner minimization). A second step amounts to minimize the resulting divergence between $P_{n}$ and $Q_{n}\left(\theta \right)$ with respect to θ (Outer minimization).

**Remark 3.**  *If $P_{0}\in$$M_{E}$ there exists an unique $P_{\theta_{0}}\in M$ such that $P_{0}=P_{\theta_{0}}\in M$; then by identifiability*

$$\arg\underset{\theta }{\inf}D_{\alpha }\left(P_{\theta },P_{\theta_{0}}\right)=\theta_{0}.$$

*In other words the unknown parameter $\theta_{0}$ is the unique minimizer of the function $D_{\alpha }\left(P_{\theta },P_{0}\right)$*

(19) $$\theta_{0}=\arg\min_{\theta }D_{\alpha }\left(P_{\theta },P_{\theta_{0}}\right)\in \Theta .$$

The empirical probability measures $P_{n}$ converge weakly a.s. to $P_{0}$ as n⟶∞. Therefore, by plugging in (19) the measures $P_{n}$ for $P_{0}$ one intuitively expects to obtain that the resulting estimator is obtained under the form

$$\arg\min_{\theta \in \Theta }M_{n}\left(P_{\theta },P_{n}\right)$$

converges to $\theta_{0}$ as n→∞, where $M_{n}\left(P_{\theta },P_{n}\right)$ is some empirical criterion which estimates the objective function $R_{\alpha }\left(P_{\theta },P_{0}\right).$

We will repeatedly make use of a basic result which we recall for convenience.

Denote $M_{n}\left(\tau \right)$ a family of random functions of a parameter τ which belongs to a space *T* endowed which a metric denoted *d*.

Assuming that the sequences $M_{n}$ converges uniformly to some deterministic function *M* defined on *T*, then the following result provides a set of sufficient conditions which entail the weak convergence of minimizers of $M_{n}$ to the minimizer of *M*, if well defined.

**Lemma 1**  ([19], Theorem 5.7)**.** *Assume that*

*(1) $\sup_{\tau \in T}\left|M_{n}\left(\tau \right)-M\left(\tau \right)\right|\overset{P}{⟶}0,$*

*(2) For any $\epsilon >0,\inf_{\left\{t\in T,d\left(t,t_{0}\right)\geq \epsilon \right\}}M\left(t\right)>M\left(t_{0}\right)$,*

*(3) the sequence $t_{n}$ satisfies*

$$M_{n}\left(t_{n}\right)\leq M_{n}\left(t_{0}\right)+\eta_{n}$$

*where $\eta_{n}=\circ_{p}\left(1\right).$ Then the sequence $t_{n}$ satisfies*

$$d\left(t_{n},t_{0}\right)\overset{P}{⟶}0.$$

Lemma 1 will be used according to the context of minimization at hand.

By (8) we consider the inner and the outer minimization problems leading to the estimator. This will be performed in two steps: the inner minimization with respect to *Q* in $M_{\theta_{E}}$ for fixed θ, and the outer minimization w.r.t θ.

Here we establish the consistency of the minimum pseudodistance estimator on the closed set of measures a.c w.r.t λ.

#### 6.1.1. Inner Minimization: Convergence of the Projection of $P_{n}$ on $M_{\theta_{E}}$

Fix θ∈Θ. Denote

$$M_{n}\left(Q\right):=R_{\alpha }\left(Q,P_{n}\right)$$

where $Q\in M_{\theta_{E}}.$

Denote

(20) $$Q_{n}\left(\theta \right):=\arg\underset{Q\in M_{\theta_{E}}}{\inf}R_{\alpha }\left(Q,P_{n}\right).$$

The existence and uniqueness of a p.m $Q_{n}\left(\theta \right)$ with density $q_{n}\left(\theta \right)$ follows from the same arguments as in Proposition 3, substituting *P* with $P_{n}$.

Denote accordingly the unique minimizer of $R_{\alpha }\left(Q,P_{0}\right)$ on $M_{\theta_{E}}$,

(21) $$q_{\theta }^{*}:=\frac{dQ_{\theta }^{*}}{dP_{0}}\;\mathrm{where}\;Q_{\theta }^{*}:=\arg\underset{Q\in M_{\theta_{E}}}{\inf}R_{\alpha }\left(Q,P_{0}\right).$$

We prove that $q_{n}\left(\theta \right)$ converges to $q_{\theta }^{*}$, making use of Lemma 1.

Setting

$$M_{n}\left(\tau \right):=R_{\alpha }\left(Q,P_{n}\right),$$

with $\tau =\frac{dQ}{d\lambda }$, setting $d\left(\tau ,\tau^{\prime }\right)=\sup_{x\in K}\left|q\left(x\right)-q^{\prime }\left(x\right)\right|$, it holds (see the proof in Appendix A).

**Lemma 2.**  *Fix θ. Then Condition (1) in Lemma 1 holds, namely*

$$\underset{Q\in M_{\theta_{E}}}{\sup}\left|R_{\alpha }\left(Q,P_{n}\right)-R_{\alpha }\left(Q,P_{0}\right)\right|\to 0\;\mathrm{in}\;\mathrm{probability}.$$

We also prove in Appendix A that the second condition in Lemma 1 holds.

**Lemma 3.**  *For any ε>0,*

$$\underset{\left\{Q:∥q-q_{\theta }^{*}∥>\epsilon ,Q\in M_{\theta_{E}}\right\}}{\inf}R_{\alpha }\left(Q,P_{0}\right)>R_{\alpha }\left(Q_{\theta }^{*},P_{0}\right).$$

*where* dQ/dP=q *and d*$Q_{\theta }^{*}/dP=q_{\theta }^{*}.$

We also state that the third condition in Lemma 1 holds.

**Lemma 4.** 
$$R_{\alpha }\left(Q_{n}\left(\theta \right),P_{n}\right)\leq R_{\alpha }\left(Q_{\theta }^{*},P_{0}\right)+\eta_{n},$$

*where $\;\eta_{n}=\circ_{p}\left(1\right).$ This follows from the very definition of $Q_{n}\left(\theta \right)$ for which $R_{\alpha }\left(Q_{n}\left(\theta \right),P_{n}\right)\leq R_{\alpha }\left(Q,P_{n}\right)$ for all $Q\in M_{\theta_{E}}.$*

Making use Lemma 1 we have proved

**Theorem 4.**  For any θ∈Θ, it holds, with $q_{n}\left(\theta \right)$ defined in (20) and $q_{\theta }^{*}$ defined in (21),

$$\underset{x\in K}{\sup}\left|q_{n}\left(\theta \right)\left(x\right)-q_{\theta }^{*}\left(x\right)\right|\overset{P}{⟶}0.$$

#### 6.1.2. Outer Minimization

We now consider the minimization in θ, with the following notation. Let

$${\hat{\theta }}_{n}:=\arg\underset{\theta }{\inf}\underset{Q\in M_{\theta_{E}}}{\inf}R_{\alpha }\left(Q,P_{n}\right)=\arg\underset{\theta }{\inf}R_{\alpha }\left(Q_{n}\left(\theta \right),P_{n}\right)$$

and

$$\theta_{0}:=\arg\underset{\theta }{\inf}\underset{Q\in M_{\theta_{E}}}{\inf}R_{\alpha }\left(Q,P_{0}\right)=\arg\underset{\theta }{\inf}R_{\alpha }\left(Q_{\theta }^{*},P_{0}\right).$$

The parameter $\theta_{0}$ such that $P_{0}=P_{\theta_{0}\;}$ is defined in a unique way by the above display; indeed, first note that $\theta_{0}$ is well defined, either when $P_{0}\in M$ (i.e., $P_{0}=P_{\theta_{0}}$) (see Theorem 1) or $P_{0}\notin M$, in which case $P_{\theta_{0}}$ is the $D_{\alpha }$-projection of $P_{0}$ on $M_{E}.$

By Theorem 4, we have proved that

$$\underset{x\in K}{\sup}\left|q_{n}\left(\theta \right)\left(x\right)-q_{\theta }^{*}\left(x\right)\right|\overset{P}{⟶}0.$$

where $q_{\theta }^{*}$ is defined in (21). We want to show that

$$\arg\underset{\theta }{\inf}R_{\alpha }\left(Q_{n}\left(\theta \right),P_{n}\right)\overset{P}{⟶}\arg\underset{\theta }{\inf}R_{\alpha }\left(Q_{\theta }^{*},P_{0}\right).$$

where $Q_{\theta }^{*}=\arg\inf_{Q\in M_{\theta_{E}}}R_{\alpha }\left(Q,P_{0}\right).$

By definition,

$${\hat{\theta }}_{n}:=\arg\underset{\theta }{\inf}R_{\alpha }\left(Q_{n}\left(\theta \right),P_{n}\right)$$

It holds

(22) $$\arg\underset{\theta }{\inf}R_{\alpha }\left(Q_{\theta }^{*},P_{0}\right)=\theta_{0}.$$

Indeed, if $P_{0}\in M$, then $P_{0}=P_{\theta_{0}}$ for some unique $\theta_{0}$ in Θ, and (22) holds.

We make use of Lemma 1 with

(23) $$\begin{array}{ccc} M_{n}\left(\theta \right) & : & =R_{\alpha }\left(Q_{n}\left(\theta \right),P_{n}\right),\\ M(\theta ) & : & =R_{\alpha }\left(Q_{\theta }^{*},P_{0}\right). \end{array}$$

Indeed, ${\hat{\theta }}_{n}$ converges to $\theta_{0}$ making use of Lemma 1.

Set $q_{n}\left(\theta \right)\left(x\right):=\frac{dQ_{n}\left(\theta \right)}{d\lambda }\left(x\right)$, and

$$d\left(q_{n}\left(\theta \right),q_{\theta }^{*}\right)=\underset{x\in K}{\sup}\left|q_{n}\left(\theta \right)\left(x\right)-q_{\theta }^{*}\left(x\right)\right|;$$

it then holds (see the proof in Appendix A).

**Proposition 4.**  *Suppose that the following condition*

(24) $$\underset{\left\{Q\in M_{\theta_{E}},Q^{\prime }\in M_{\theta_{E}^{\prime }},d\left(\theta ,\theta^{\prime }\right)<\delta \right\}}{\sup}d\left(q,q^{\prime }\right)<C\delta$$

*holds for some C>0 independent on θ and $\theta^{\prime }$; then*

$$\underset{\theta \in \Theta }{\sup}\underset{x\in K}{\sup}\left|q_{n}\left(\theta \right)\left(x\right)-q_{\theta }^{*}\left(x\right)\right|\overset{P}{⟶}0.$$

**Lemma 5.**  *Under Condition (24) in Proposition 4, condition (1) in Lemma 1 holds, i.e.,*

$$\underset{\theta \in \Theta }{\sup}\left|M_{n}\left(\theta \right)-M\left(\theta \right)\right|\overset{P}{⟶}0$$

*with $M_{n}\left(\theta \right)$ and M(θ) defined in (23).*

We now state that the second condition in Lemma 1 holds (see the proof in Appendix A).

**Lemma 6.**  *For any ε>0, $\inf_{|\theta -\theta_{0}|>\epsilon }M\left(\theta \right)>M\left(\theta_{0}\right)$.*

We also state the third condition in Lemma 1 (see the proof in Appendix A).

**Lemma 7.**  *$M_{n}\left(\theta \right)\leq M\left(\theta \right)+\circ_{p}\left(1\right)$.*

As a consequence of the above arguments, the following convergence result for the minimization of power type divergences on semiparametric models defined by moment conditions holds.

**Theorem 5.**  *Under all the above conditions (11)–(14), (16) and (24), it holds, whenever $P_{0}$ belongs to M or $P_{0}$ belongs to $M_{E},$ with corresponding $\theta_{0},$*

$$\lim_{n\to \infty }D_{\alpha }\left(M,P_{n}\right)=0$$

*and*

$$\lim_{n\to \infty }{\hat{\theta }}_{n}=\theta_{0}$$

*Also, we get*

$$\lim_{n\to \infty }d\left(q_{\hat{\theta_{n}}},p_{\theta_{0}}\right)=0$$

*and all convergences above hold in probability.*

**Remark 4.**  *Note that a sufficient condition for the existence and uniqueness of the projection of $P_{0}$ on M is obtainable under a weaker condition than (12); however, (12) implies that E is a Donsker class (hence a Glivenko–Cantelli class), which is a convenient argument for the convergence of the estimator; in the same vein, this Donsker property should clearly hold for the asymptotic distribution. Therefore, (12) seems a suitable nearly unavoidable assumption.*

### 6.2. A Remark on the Asymptotic Distribution of the Estimate

By its very nature the $D_{\alpha }$ divergence is suited to statistical inference in a strictly parametric setting, as is the modified Kullback–Leibler divergence, whose minimization amounts to maximum likelihood. Recall that both coincide when α=0. In the case when α=0, the likelihood estimating equation, assuming $P_{\theta }$ with density $p_{\theta }$, is written

(25) $$\sum_{i=1}^{n}\overset{´}{l}\left(X_{i}\right):=\sum_{i=1}^{n}{\left(\frac{d}{d\theta }\log p_{\theta }\left(X_{i}\right)\right)}_{{\hat{\theta }}_{n}}=0$$

where ${\hat{\theta }}_{n}$ denotes the MLE, under suitable regularity conditions.

Recall the general scheme leading to the asymptotic distribution of estimators adapted to the present context: assume that the distribution $P_{\theta_{0}}$ of the data, denoted $P_{\theta_{0},q_{0}}$ (with $q_{0}:=dP_{\theta_{0}}/d\lambda$), belongs to the model $M_{E}$ and is embedded in a class of distributions $P_{\theta ,q}$ with θ∈Θ and q∈H, a Hilbert space of functions defined on *K* which contains E. A classical method amounts to substituting the classical score $\overset{´}{l}=$$\frac{d}{d\theta }\log\;p_{\theta }$ in the estimating Equation (25) by the efficient score ${\tilde{l}}_{\theta ,q}$, where ${\tilde{l}}_{\theta ,q}\left(x\right):={\overset{´}{l}}_{\theta ,q}\left(x\right)-\Pi_{\theta ,q}($${\overset{´}{l}}_{\theta ,q})$ where ${\overset{´}{l}}_{\theta ,q}\left(x\right)$ denotes the parametric score function in the semiparametric model $P_{\theta ,q}$ for θ when *q* is fixed and $\Pi_{\theta ,q}$ is the orthogonal projection onto the closure of the nuisance score space for *q*. The Influence function of the resulting efficient estimator ${\hat{\theta }}_{n}$ is ${\tilde{l}}_{\theta_{0},q_{0}}$, which yields the asymptotic Gaussian approximation for $\sqrt{n}$$\left({\hat{\theta }}_{n}-\theta_{0}\right)$, with asymptotic covariance matrix ∫${\tilde{l}}_{\theta_{0},q_{0}}^{T}$${\tilde{l}}_{\theta_{0},q_{0}}dP_{\theta_{0},q_{0}}.$ We refer to [19] for a precise account and examples, with explicit techniques for the estimation of the asymptotic covariance of the estimator.

In our context, the estimator ${\hat{\theta }}_{n}$ results from the two-step optimization scheme, defined as inner optimization which for any θ provides $q_{n}\left(\theta \right)$ in $M_{E_{\theta }}$ and the outer optimization which yields ${\hat{\theta }}_{n}.$ The case when α∈(0,1] may be considered following a similar approach as in the case α=0, but the description of the nuisance score space is somehow more involved, since it amounts to considering the set of differentials of the sub model $t\to P_{\theta_{t},q\left(\theta_{t}\right)\;}$ with $P_{\theta_{0},q\left(\theta_{0}\right)}=P_{\theta_{0},q_{0}}$ along regular paths at t=0, and to obtaining the Influence function of ${\hat{\theta }}_{n}$ through projection. This two-step procedure has been considered in the econometric literature in the context of moment constrained optimization (of regression type) with functional nuisance parameter; see [20,21,22] and references therein. A convenient approach consists in approximating elements in the nuisance space H by finite dimensional vectors (for examples by sieves); see, e.g., [23] for explicit treatment.

A description of those asymptotics in the context of regression semiparametric models is postponed to a future work.

## 7. Estimating with Polynomials

A very simple toy case illustrates the present approach; consider a class of polynomials $p\left(x\right)=ax^{2}$+bx+c on $\left[0,1\right]$ which take positive values on $\left[0,1\right].$ Let $p_{0}\left(x\right)$ satisfy $\int_{0}^{1}p_{0}\left(x\right)dx=1$, a=4, and $\int_{0}^{1}xp_{0}\left(x\right)dx=\mu =0.4.$ The resulting polynomial is written

$$p_{0}\left(x\right)=4x^{2}-\frac{26}{5}x+\frac{34}{15}.$$

The corresponding polynomial is positive on R.

Now choose α=0.5. Let *E* be the class of polynomials with coefficients close to those of $p_{0}$ and such that both (11) and (12) hold with δ=α (all elements in *E* are bounded Lipschitz functions on $\left[0,1\right]$). For example, we may consider *E* the class of polynomials with degree 2 of the form $4x^{2}+bx+c$ with $b^{2}-16c<0$, which entails (12) (i), and therefore adequacy holds for all α in (0,1]. Regularity of elements in this class guarantees this latest assertion. Therefore, adequacy holds between $D_{\alpha }$ and M, which is defined through l=1, g(x,μ)=x−μ and the class E. In the present toy case, any value of α in (0,1] allows for adequacy.

Conditions (13), (14) and (24) are easily verified, with $\theta =\mu \in \left(u,v\right)∋0.4$, since in this elementary example, the polynomial *p* has only one degree of freedom, as a function of μ. Also, (24) trivially holds since $\inf_{Q\in M_{0.4}}D_{\alpha }($$Q,P_{0})=0$ where $dP_{0}/d\lambda =p_{0}.$ Therefore, adequacy holds.

We simulate *n* points with density $p_{0}$ and choose α=1/2. The aim of this exercise is to recover estimates of *a* and μ.

**Figure 1:**
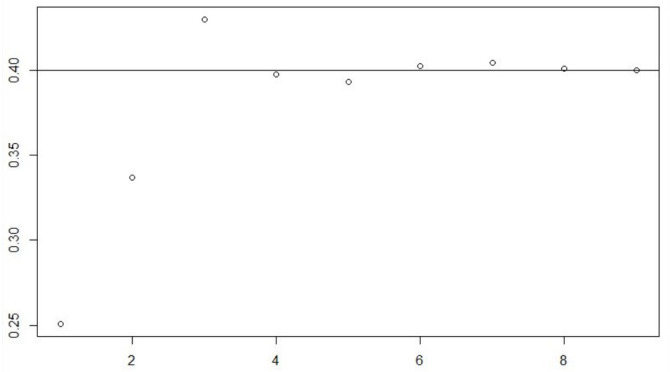
Estimation of the parameter μ for *n* = 10, 50, 100, 500, 1000, 5000, 10,000, 50,000, 100,000. The abscissa quotes 2 for *n* = 50, 4 for *n* = 500, 6 for *n* = 5000, 8 for *n* = 50,000.

**Figure 2:**
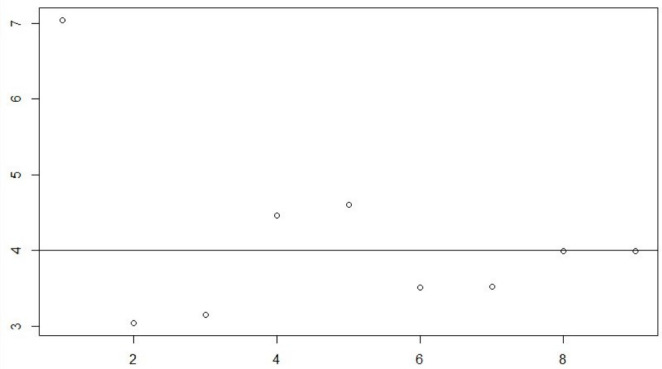
Estimation of the coefficient *a* for *n* = 10, 50, 100, 500, 1000, 5000, 10,000, 50,000, 100,000. The abscissa quotes 2 for *n* = 50, 4 for *n* = 500, 6 for *n* = 5000, 8 for *n* = 50,000.

The problem at hand here is therefore to find the value of (a,μ). For the estimation of μ solve

$$\hat{\mu }=\arg\min_{\mu \in \Theta }\min_{Q\in M_{\mu_{E}}}R_{\alpha }\left(Q,P_{n}\right)$$

where $P_{n}$ will be obtained by sampling with the true parameters, for a given sample size *n*. For any running value of μ, say $\mu_{k}$, the minimization of $R_{\alpha }\left(Q,P_{n}\right)$ with respect to all polynomials *q* with degree less than or equal 2, with integral 1, with positive values on $\left[0,1\right]$, and satisfying $\int_{0}^{1}xq\left(x\right)dx=\mu_{k}$ provides $Q_{k}$ in $M_{\mu_{k}E}$, hence the inner optimization. Evaluation of $R_{\alpha }\left(Q_{k},P_{n}\right)$ on a grid of values $\mu_{k}$ provides the outer optimization. Figure 1 and Figure 2 hereunder capture the results; in Figure 2, we quote the estimate of *a*, since both constraints $\int_{0}^{1}q\left(x\right)dx=1$ and $\int_{0}^{1}xq\left(x\right)dx=\mu$ provide *q* for given a.

## 8. Conclusions

Adding smoothness to moment constrained models introduces the need for adequate inferential techniques; indeed, the context underlying the fact that the omnibus $L^{2}$ and Kullback–Leibler divergences are the only valid ones for models defined through moment constraints, as discussed in [8], fall short under additional regularity requirements. This introduces the need for divergence-based approaches under alternative pseudo-distances.

In this paper we have stated a set of regularity conditions pertaining to a smooth moment constrained model indexed by a finite dimensional parameter of interest θ and a functional nuisance parameter *q* in some function class *E* (which is the space of smooth densities of the model); those allow for adequacy with some power divergences $D_{\alpha }$ with 0<α≤1; those conditions firstly validate the choice of $D_{\alpha }$ for the inference through their analytical properties (implying existence and uniqueness of $D_{\alpha }$-projections on the model). Furthermore, they entail consistency of the estimators. Condition (12) appears as a good compromise between analytic and statistical requirements, which we call adequacy. The two-step optimization procedure produces consistent estimators of both true parameters $\theta_{T}$ and $q_{T}$.

Adequacy holds as follows:

Either the class *E* is lower bounded, and $q^{\delta }$ is Lipschitz uniformly over *E* for some $\delta \in \left(0,1\right]$ or the class *E* cannot be uniformly lower bounded on *K* but $q^{\delta }$ is Lipschitz uniformly over *E* for some δ:0<δ<α.

Additionally adequacy requires sharp requirements which enforce identifiability, mainly strong separation between the submodels indexed by θ (see (13), (14), (16) and (24)); condition (16) establishes a connection between the structure of the model and the divergence.

Other semiparametric models of the same type frequently occur in the econometric or reliability literature, for example, when the nuisance parameter consists in subsets of regular convex or monotone bounded densities (see, e.g., [24] Chapter 3), or models with restricted hazard rate functions.

The limit distributions of the couple of parameters $\theta_{T}$ and $q_{T}$ are not handled in this paper and can be studied making use of now classical semiparametric inferential methods (see [19,23]); however, due to the two-step framework of our optimization proposal, it could be wise to consider approximating schemes (sieves or RKHS) for the nuisance parameter space. This is postponed to future work.

A final remark addresses the applicability of the present approach for large (or even high) dimensional models; high dimensional sieves can be introduced in order to approximate the assumed regularity of the model. In those large dimensional settings minimum divergence inference is based on gradient descent techniques; see also a Monte Carlo approach in [25]. Some alternative approaches have been proposed in the multivariate case; see, for example, [26,27] making use of skewness maximization to obtain the linear projection maximizing the divergence from a normal distribution under a semiparametric setting, where the sampled distribution is assumed to be a finite mixture of symmetric distribution with finite third moments.

## Data Availability

The original contributions presented in this study are included in the article.

## Appendix A

### Appendix A.1. Proof of Theorem 1

First case: Suppose that $P_{0}=P_{\theta_{0}}\in M_{E}$, i.e., $P_{0}$$\in M_{\theta_{0_{E}}}.$ Then

$$\underset{Q\in M_{\theta_{0_{E}}}}{\inf}D_{\alpha }\left(Q,P_{0}\right)=0.$$

Since $M_{E}⊃M_{\theta_{0_{E}}}$, we have

$$\underset{Q\in M_{E}}{\inf}D_{\alpha }\left(Q,P_{0}\right)=0.$$

Furthermore, $\theta_{0}$ realizes $\inf_{Q\in M_{\theta_{0_{E}}}}D_{\alpha }\left(Q,P_{0}\right)=0$.

So

$$\theta_{0}\in \arg\underset{\theta }{\inf}\underset{Q\in M_{\theta_{0_{E}}}}{\inf}D_{\alpha }\left(Q,P_{0}\right).$$

We prove that $\theta_{0}$ is the only parameter θ that satisfies $\inf_{Q\in M_{\theta_{0_{E}}}}D_{\alpha }\left(Q,P_{0}\right)=0$.

Suppose that $\theta_{1}\neq \theta_{0}$ such that $\theta_{1}\in \arg\inf_{\theta }\inf_{Q\in M_{\theta_{0_{E}}}}D_{\alpha }\left(Q,P_{0}\right)$.Then

$$\underset{Q\in M_{\theta_{1_{E}}}}{\inf}D_{\alpha }\left(Q,P_{0}\right)=\underset{Q\in M_{\theta_{0_{E}}}}{\inf}D_{\alpha }\left(Q,P_{0}\right)=0.$$

Since $M_{\theta_{1_{E}}}\subset M_{\theta_{1}}$

$$0=\underset{Q\in M_{\theta_{1_{E}}}}{\inf}D_{\alpha }\left(Q,P_{0}\right)\geq \underset{Q\in M_{\theta_{1}}}{\inf}D_{\alpha }\left(Q,P_{0}\right)\geq 0.$$

Hence,

$$\underset{Q\in M_{\theta_{1}}}{\inf}D_{\alpha }\left(Q,P_{0}\right)=0.$$

Therefore, $\theta_{0}$ is the only θ such that $P_{0}\in M_{\theta_{0}}$, which proves that $\theta_{1}$ does not exist (otherwise $P_{0}=P_{\theta_{1}}$ due to (13).

Second case: Suppose that $\;\;P_{0}=P_{\theta_{0}}\in M$ and $P_{0}\notin M_{E}.$ Recall that

$$\theta_{0}=\arg\underset{\theta }{\inf}\underset{Q\in M_{\theta }}{\inf}D_{\alpha }\left(Q,P_{0}\right).$$

We now show that

$$\theta_{0}=\arg\underset{\theta }{\inf}\underset{Q\in M_{\theta_{E}}}{\inf}D_{\alpha }\left(Q,P_{0}\right).$$

Project $P_{0}=P_{\theta_{0}}$ on $M_{E}$ and define

$$\theta_{1}\in \arg\underset{\theta }{\inf}\underset{Q\in M_{\theta_{E}}}{\inf}D_{\alpha }\left(Q,P_{0}\right).$$

Assume that $\theta_{1}\neq \theta_{0}$.

We then have

$$\underset{Q\in M_{\theta_{1_{E}}}}{\inf}D_{\alpha }\left(Q,P_{0}\right)\leq \underset{Q\in M_{\theta_{E}}}{\inf}D_{\alpha }\left(Q,P_{0}\right)$$

for all θ by definition of $\theta_{1}$. So with $\theta =\theta_{0}$, we have

(A1) $$\underset{Q\in M_{\theta_{1_{E}}}}{\inf}D_{\alpha }\left(Q,P_{0}\right)\leq \underset{Q\in M_{\theta_{0_{E}}}}{\inf}D_{\alpha }\left(Q,P_{0}\right).$$

Under (16) it holds $D_{\alpha }\left(M_{\theta_{0_{E}}},P_{\theta_{0}}\right)<D_{\alpha }\left(M_{\theta_{E}},P_{\theta_{0}}\right)$, for all $\theta \neq \theta_{0}$.

Hence, (A1) is impossible, so $\theta_{1}=\theta_{0}$. We have proved (17).

### Appendix A.2. Proof of Theorem 2

Assume that ${\left(Q_{n}\right)}_{n\geq 1}\subset M_{E}$ and assume that there exists *q* such that

$$\underset{x}{\sup}\left|q_{n}\left(x\right)-q\left(x\right)\right|⟶0,$$

with $q_{n}\left(x\right):=\left(dQ_{n}/d\lambda \right)\left(x\right).$ Define $Q\left(A\right):=\int_{A}q\left(x\right)d\lambda \left(x\right)$ for any set *A* and we have $\left(Q_{n}\underset{st}{⟶}Q\right)$. We prove that $Q\in M_{E},$ stating

(A) *q* is a density;

(B) $\;\int_{K}g\left(x,\theta \right)q\left(x\right)dx=0$ for some θ;

(C) *q* satisfies (11) and (12).

We prove (A); this follows from the Prohorov Theorem.

We prove (B). Let $\theta_{n}$ be defined by $\int g\left(x,\theta_{n}\right)q_{n}\left(x\right)dx=0;$ such that $\theta_{n}$ indeed exists since $Q_{n}\in M$.

Since Θ is a compact set in $R^{d}$, we select $n_{j}\subset n$ such that the subsequence $\theta_{n_{j}}$ admits a limit $\underline{\theta }$ and $\int g\left(x,\theta_{n_{j}}\right)q_{n_{j}}\left(x\right)dx=0$.

We prove that $\left|\int_{K}g\left(x,\underline{\theta }\right)q\left(x\right)dx\right|=0$.

Indeed,

$$\begin{array}{cc} \left|\int_{K}g\left(x,\underline{\theta }\right)q\left(x\right)dx\right| & \leq \left|\int_{K}g\left(x,\underline{\theta }\right)q_{n_{j}}\left(x\right)dx\right|+\left|\int_{K}g\left(x,\underline{\theta }\right)q\left(x\right)dx-\int_{K}g\left(x,\underline{\theta }\right)q_{n_{j}}\left(x\right))dx\right|\\ \; & \leq B+A \end{array}$$

$$A=\left|\int_{K}g\left(x,\underline{\theta }\right)\left(q\left(x\right)-q_{n_{j}}\left(x\right)\right)dx\right|$$

which tends to 0 by (10).

Next

$$B\leq \int_{K}\left|g\left(x,\underline{\theta }\right)-g\left(x,\theta_{n_{j}}\right)\right|q_{n_{j}}\left(x\right)dx+\left|\int_{K}g\left(x,\theta_{n_{j}}\right)q_{n_{j}}\left(x\right)dx\right|\leq C+D$$

and D=0 by definition of $\theta_{n_{j}}$.

Hence,

$$\begin{array}{ccc} B & \leq & C=\int_{K}\left|g\left(x,\underline{\theta }\right)-g\left(x,\theta_{n_{j}}\right)\right|q_{n_{j}}\left(x\right)dx\\ & \leq & \underset{x\in K}{\sup}\left|g\left(x,\underline{\theta }\right)-g\left(x,\theta_{n_{j}}\right)\right|\int_{K}q_{n_{j}}\left(x\right)dx\\ & = & \underset{x\in K}{\sup}\left|g\left(x,\underline{\theta }\right)-g\left(x,\theta_{n_{j}}\right)\right|\to 0. \end{array}$$

We have proved that any converging sequence $\theta_{n_{j}}$ satisfies $\int_{K}g\left(x,\underline{\theta }\right)q\left(x\right)dx$ when $\underline{\theta }=\lim_{n_{j}\to \infty }\theta_{n_{j}}$.

Consider two converging subsequences $n_{j}$ and $n_{j}^{\prime }$ with $\theta_{n_{j}}\to \underline{\theta }$ and $\theta_{n_{j}}^{\prime }\to \bar{\theta }$; we have

$$\int_{K}g\left(x,\underline{\theta }\right)q\left(x\right)dx=\int_{K}g\left(x,\bar{\theta }\right)q\left(x\right)dx.$$

By (13) it follows that $\underline{\theta }=\bar{\theta }$; therefore, we have proved that there exists a unique θ∈Θ such that

$$\int_{K}g\left(x,\theta \right)q\left(x\right)dx=0$$

which proves (B).

We prove (C); firstly there exists some N>0 such that $|q(x_{0})|\leq N$. Indeed,

$$|q\left(x_{0}\right)-q_{n}\left(x_{0}\right)+q_{n}\left(x_{0}\right)\left|\leq \right|q_{n}\left(x_{0}\right)\left|+\right|q_{n}\left(x_{0}\right)-q\left(x_{0}\right)\left|\leq N+\right|q_{n}\left(x_{0}\right)-q\left(x_{0}\right)|\leq N+\varepsilon$$

for all ε>0 and therefore $|q(x_{0})|\leq N$, since

$$|q_{n}\left(x_{0}\right)-q\left(x_{0}\right)|\to 0.$$

This proves (11). We prove that *q* satisfies (12).

Assume that there exists $\left(x,y\right)$ in *K* such that $\left|q^{\alpha }\left(x\right)-q^{\alpha }\left(y\right)\right|>M\left|x-y\right|.$ By the triangle inequality it then holds

$$\left|q^{\alpha }\left(x\right)-q^{\alpha }\left(y\right)\right|\leq \left|q_{n}^{\alpha }\left(x\right)-q_{n}^{\alpha }\left(y\right)\right|+2\varepsilon_{n}$$

where $\varepsilon_{n}:=\sup_{x\in K}\left|q^{\alpha }\left(x\right)-q_{n}^{\alpha }\left(x\right)\right|\to 0$, whence $\left|q_{n}^{\alpha }\left(x\right)-q_{n}^{\alpha }\left(y\right)\right|>M\left|x-y\right|+\varepsilon_{n}^{\prime }$ with $\varepsilon_{n}^{\prime }$→0, a contradiction. Now either (12) (i) or (12) (ii) clearly hold.

### Appendix A.3. Proof of Proposition 2

We prove that $A_{E}\left(a\right)$ is a closed subset in $M_{E}$ equipped with the strong topology. Recall that $Q\to D_{\alpha }\left(Q,P\right)$ l.s.c is equivalent to $A_{E}\left(a\right)$, which is closed.

Let $Q_{n}\in A_{E}\left(a\right)\cap M_{E}$. Denote $\frac{dQ_{n}}{d\lambda }\left(x\right)=q_{n}\left(x\right)$ with $q_{n}\in E$, and assume that there exists a function *q* defined on *K* such that

$$\underset{x\in K}{\sup}\left|q_{n}\left(x\right)-q\left(x\right)\right|\to 0.$$

Define

$$\frac{dQ}{d\lambda }\left(x\right)=q\left(x\right).$$

We prove that q∈E and that $Q\in A_{E}\left(a\right)$ with $Q\left(A\right):=\int 1_{A}\left(x\right)q\left(x\right)d\lambda \left(x\right)$.

Since $M_{E}$ is closed, (see Theorem 2) the measure *Q* defined by $Q\left(A\right)=\int 1_{A}\left(x\right)q\left(x\right)d\lambda \left(x\right)$ for all A∈B(R) is in $M_{E}$.

It remains to prove that $D_{\alpha }\left(Q,P\right)\leq a$.

It holds $\sup_{x\in K}\left|q_{n}^{\beta }\left(x\right)-q^{\beta }\left(x\right)\right|\to 0$ for all β>0.

Consider now the mapping

$$x\to \varphi \left(q_{n}\left(x\right),p\left(x\right)\right)-\varphi \left(q\left(x\right),p\left(x\right)\right).$$

Since

$$\varphi \left(q_{n}\left(x\right),p\left(x\right)\right)-\varphi \left(q\left(x\right),p\left(x\right)\right)=q_{n}^{\alpha +1}\left(x\right)-q^{\alpha +1}\left(x\right)-\left(1+\frac{1}{\alpha }\right)p\left(x\right)\left(q_{n}^{\alpha }\left(x\right)-q^{\alpha }\left(x\right)\right);$$

it holds

$$\underset{x\in K}{\sup}\left|\varphi \left(q_{n}\left(x\right),p\left(x\right)\right)-\varphi \left(q\left(x\right),p\left(x\right)\right)\right|\to 0.$$

Integrating, we have

(A2) $$\int \varphi \left(q_{n}\left(x\right),p\left(x\right)\right)dx-\delta \leq \int \varphi \left(q\left(x\right),p\left(x\right)\right)dx=D_{\alpha }\left(Q,P\right)\leq \int \varphi \left(q_{n}\left(x\right),p\left(x\right)\right)dx+\delta$$

for any δ>0, for large *n*. Since $Q_{n}\in A_{E}\left(a\right),\int \varphi \left(q_{n}\left(x\right),p\left(x\right)\right)dx\leq a$, the inequality (A2) becomes

$$\begin{array}{cc} \int \varphi \left(q_{n}\left(x\right),p\left(x\right)\right)dx-\delta \leq \int \varphi \left(q\left(x\right),p\left(x\right)\right)dx\leq \int \varphi \left(q_{n}\left(x\right),p\left(x\right)\right)dx+\delta & \leq \\ \; & a+\delta \end{array}$$

So ∫φ(q(x),p(x))dx≤a; hence, $Q\in A_{E}\left(a\right)$ and thus $A_{E}\left(a\right)$ is a closed set in $M_{E}$.

### Appendix A.4. Proof of Proposition 3

The set *F* of functions *q* in *E* such that $D_{\alpha }\left(Q,P\right)\leq a$ for *Q* with density *q* is closed in *E* by Proposition 2; now cl(E) is compact by the Arzela-Ascoli Theorem; hence, *F* is a compact subset in *E*.

The mapping q→Q from *E* to $M_{E}$ is injective, whence $A_{E}\left(a\right)$ is compact in $M_{E}.$ Let $a_{\theta }:=\inf_{Q\in M_{\theta_{E}}}D_{\alpha }\left(Q,P\right)$ and let ε>0. Then $A_{E}\left(a_{\theta }+\varepsilon \right)\cap M_{\theta_{E}}\neq \emptyset .$

It can be observed that for all θ the set $M_{\theta_{E}}$ is a closed set, following the same arguments as in Proposition 2. Since $M_{\theta_{E}}$ is closed and $A_{E}\left(a_{\theta }+\varepsilon \right)$ is compact, $A_{E}\left(a_{\theta }+\varepsilon \right)\cap M_{\theta_{E}}$ is compact.

Since

$$\arg\underset{Q\in M_{\theta_{E}}}{\inf}D_{\alpha }\left(Q,P\right)=\arg\underset{Q\in A_{E}\left(a_{\theta }+\varepsilon \right)\cap M_{\theta_{E}}}{\inf}D_{\alpha }\left(Q,P\right),$$

the existence of the projection follows from the lower semi-continuity of $Q\to D_{\alpha }\left(Q,P\right).$ Since φ is strictly convex the function $Q\in M_{K}^{1}\left(\lambda \right)\to D_{\alpha }\left(Q,P\right)$ is also strictly convex, and the projection of *P* on any closed convex set Ω in $M_{\theta_{E}}$ is uniquely defined.

### Appendix A.5. Proof of Lemma 2

It holds

$$\underset{Q\in M_{\theta_{E}}}{\sup}\left|R_{\alpha }\left(Q,P_{n}\right)-R_{\alpha }\left(Q,P_{0}\right)\right|\leq \left(1+\frac{1}{\alpha }\right)\underset{Q\in M_{\theta_{E}}}{\sup}\left|\frac{1}{n}\sum_{i=1}^{n}q^{\alpha }\left(X_{i}\right)-E_{P_{0}}\left(q^{\alpha }\left(X\right)\right)\right|$$

which tends to 0 almost surely as *n* tends to infinity; indeed, we may use the arguments developed in [24] (pp. 154–157), making use of condition (12), which implies, in its notation, that the class of function $x\to q^{\alpha }$ belongs to $C_{M}^{\alpha }\left(K\right)$; therefore, the class *E* is Donsker, by Corollary 2.7.2. in [24], and henceforth is a Glivenko–Cantelli class.

### Appendix A.6. Proof of Lemma 3

We thus prove Condition (2) in Lemma 1. By Proposition 3

$$Q_{\theta }^{*}:=\arg\underset{Q\in M_{\theta_{E}}}{\inf}R_{\alpha }\left(Q,P_{0}\right)$$

exists with uniqueness. Denote $q_{\theta }^{*}:=\frac{dQ^{*}\left(\theta \right)}{d\lambda }$. It holds

$$\underset{∥q-q_{\theta }^{*}∥>\varepsilon ,q\in M_{\theta_{E}}}{\inf}R_{\alpha }\left(Q,P_{0}\right)>R_{\alpha }\left(Q^{*}\left(\theta \right),P_{0}\right).$$

Indeed, by definition for all *Q*, such that $\frac{dQ}{d\lambda }\left(x\right)=q\left(x\right)$

$$R_{\alpha }\left(Q^{*}\left(\theta \right),P_{0}\right)\leq R_{\alpha }\left(Q,P_{0}\right)$$

and therefore,

$$\;R_{\alpha }\left(Q^{*}\left(\theta \right),P_{0}\right)\leq \underset{∥q-q^{*}\left(\theta \right)∥>\varepsilon }{\inf}R_{\alpha }\left(Q,P_{0}\right).$$

Now let $Q^{*}\left(\theta \right)$ and denote $dQ^{*}\left(\theta \right)/d\lambda \left(x\right)=q^{*}\left(\theta \right)\left(x\right)$ and *Q* such that dQ(θ)/dλ(x)=q(θ)(x). We prove that the inequality is strict. From the above display we get

$$R_{\alpha }\left(Q^{*}\left(\theta \right),P_{0}\right)+\frac{1}{\alpha }\int p_{0}^{\alpha +1}\left(x\right)dx\leq \underset{∥q-q^{*}\left(\theta \right)∥>\varepsilon }{\inf}\left\{R_{\alpha }\left(Q,P_{0}\right)+\frac{1}{\alpha }\int p_{0}^{\alpha +1}\left(x\right)dx\right\}$$

i.e.,

$$D_{\alpha }\left(M_{\theta_{E}},P_{0}\right)\leq \underset{∥q-q^{*}\left(\theta \right)∥>\varepsilon ,q\in M_{\theta_{E}}}{\inf}D_{\alpha }\left(Q,P_{0}\right).$$

Now if equality holds, there exists $q\in M_{\theta_{E}}$, $q\neq q^{*}\left(\theta \right)$ such that

(A3) $$D_{\alpha }\left(M_{\theta_{E}},P_{0}\right)=D_{\alpha }\left(Q^{*}\left(\theta \right),P_{0}\right)=D_{\alpha }\left(Q,P_{0}\right).$$

It holds $Q\neq Q^{*}\left(\theta \right)$ since both $q^{*}\left(\theta \right)$ and q∈E. But the projection of $P_{0}$ on $M_{\theta_{E}}$ is unique, so (A3) cannot hold.

### Appendix A.7. Proof of Proposition 4

By Theorem 4 for all θ

$$d(q_{n}\left(\theta \right),q_{\theta }^{*})\to 0\;\mathrm{in}\;\mathrm{probability}.$$

We want to prove that uniform convergence upon θ holds. Define $\theta_{n}$ by

(A4) $$sup_{\theta \in \Theta }d\left(q_{n}\left(\theta \right),q_{\theta }^{*}\right)=d\left(q_{n}\left(\theta_{n}\right),q_{\theta_{n}}^{*}\right).$$

Let $\left\{n_{j}\right\}\subset \left\{n\right\}$ and suppose $\underline{\theta }$ such that $\theta_{n_{j}}\to \underline{\theta }.$

We show that $d(q_{n_{j}}\left(\theta_{n_{j}}\right),q_{\theta_{n_{j}}}^{*})>c>0$ cannot hold.

By definition (A4)

$$\begin{array}{ccc} sup_{\theta \in \Theta }d\left(q_{n_{j}}\left(\theta \right),q_{\theta }^{*}\right) & = & d(q_{n_{j}}\left(\theta_{n_{j}}\right),q_{\theta_{n_{j}}}^{*})\\ & \leq & d\left(q_{n_{j}}\left(\theta_{n_{j}}\right),q_{n_{j}}\left(\underline{\theta }\right)\right)+d\left(q_{n_{j}}\left(\underline{\theta }\right),q_{\underline{\theta }}^{*}\right)+d\left(q_{\theta_{n_{j}}}^{*},q_{\underline{\theta }}^{*}\right)\\ & = & :I_{1}+I_{2}+I_{3}. \end{array}$$

Now $I_{1}=d\left(q_{n_{j}}\left(\theta_{n_{j}}\right),q_{n_{j}}\left(\underline{\theta }\right)\right)$ and $d(\theta_{n_{j}},\underline{\theta })\to 0.$ Hence under (24), $I_{1}\overset{P}{⟶}0.$ Now $I_{2}=d\left(q_{n_{j}}\left(\underline{\theta }\right),q_{\underline{\theta }}^{*}\right)$; both $q_{n_{j}}\left(\underline{\theta }\right)$ and $q_{\underline{\theta }}^{*}$ belong to $M_{{\underline{\theta }}_{E}}$; By Theorem 4 in $M_{{\underline{\theta }}_{E}}$, $d\left(q_{n_{j}}\left(\underline{\theta }\right),q_{\underline{\theta }}^{*}\right)\overset{P}{⟶}0$ so $I_{2}\overset{P}{⟶}0,$

As for $I_{3}=d\left(q_{\underline{\theta }}^{*},q_{\theta_{n_{j}}}^{*}\right)\overset{P}{⟶}0$, as for $I_{1}.$ We have proved that

(A5) $$\lim_{j\to \infty }\underset{\theta \in \Theta }{\sup}d\left(q_{n_{j}}\left(\theta \right),q_{\theta }^{*}\right)=0\;\mathrm{in}\;\mathrm{probability}.$$

Assume now that (A4) does not hold. In such a case there exists a subsequence $\left\{m_{k}\right\}\subset \left\{n\right\}$ and η>0 such that

$$\underset{\theta }{\sup}d\left(q_{m_{k}}\left(\theta \right),q_{\theta }^{*}\right)>\eta .$$

Let $\theta_{m_{k}}:=\arg\sup_{\theta }d\left(q_{m_{kj}}\left(\theta \right),q_{\theta }^{*}\right)$, whence

$$d(q_{m_{k}}\left(\theta_{n_{j}}\right),q_{\theta_{m_{k}}}^{*})>\eta$$

for all *k*. Extract from $\left\{m_{k}\right\}$ a further subsequence $\left\{n_{j}\right\}$ along which $\theta_{n_{j}}$ converges to some $\underline{\theta }.$ Then (A5) proves our claim, by contradiction.

### Appendix A.8. Proof of Lemma 5

Define

$$M_{n}\left(\theta \right)=R_{\alpha }\left(q_{n}\left(\theta \right),P_{n}\right),\;\mathrm{and}\;M\left(\theta \right)=R_{\alpha }\left(q_{\theta }^{*},P_{0}\right)$$

with

$$R_{\alpha }\left(q_{n}\left(\theta \right),P_{n}\right)=\int q_{n}^{\alpha +1}\left(\theta \right)\left(x\right)dx-\left(1+\frac{1}{\alpha }\right)\int q_{n}^{\alpha }\left(\theta \right)\left(x\right)dP_{n}\left(x\right)$$

and

$$R_{\alpha }\left(q_{\theta }^{*},P_{0}\right)=\int q_{\theta }^{*\alpha +1}\left(x\right)dx-\left(1+\frac{1}{\alpha }\right)\int q_{\theta }^{*\alpha }\left(x\right)dP_{0}\left(x\right).$$

Hence,

$$\begin{array}{cc} \underset{\theta \in \Theta }{\sup}\left|M_{n}\left(\theta \right)-M\left(\theta \right)\right| & \leq \underset{\theta \in \Theta }{\sup}\int \left|q_{n}^{\alpha +1}\left(\theta \right)\left(x\right)-q_{\theta }^{*\alpha +1}\left(x\right)\right|dx+\\ \; & \left(1+\frac{1}{\alpha }\right)\underset{\theta \in \Theta }{\sup}\int \left|q_{n}^{\alpha }\left(\theta \right)\left(x\right)-q_{\theta }^{*\alpha }\left(x\right)\right|dP_{n}\left(x\right)\\ \; & +\left(1+\frac{1}{\alpha }\right)\underset{\theta \in \Theta }{\sup}\left|\int q_{\theta }^{*\alpha }\left(x\right)d\left(P_{n}-P_{0}\right)\right|\\ \; & \leq R_{1}+R_{2}+R_{3}. \end{array}$$

Now

$$\begin{array}{ccc} R_{1} & = & \underset{\theta \in \Theta }{\sup}\int \left|q_{n}^{\alpha +1}\left(\theta \right)\left(x\right)-q_{\theta }^{*\alpha +1}\left(x\right)\right|dx\\ & \leq & \underset{\theta \in \Theta }{\sup}\underset{x\in K}{\sup}\left|q_{n}\left(\theta \right)\left(x\right)-q_{\theta }^{*}\left(x\right)\right|\times C_{1} \end{array}$$

which tends to 0 in probability by Proposition 4. The constant $C_{1}$ in the above inequality is $\left(\alpha +1\right)\max(\sup_{x\in K}q_{n}^{\alpha }\left(\theta \right)\left(x\right),\sup_{x\in K}q_{\theta }^{*\alpha }\left(x\right))$, which is bounded uniformly in *n*.

Also,

$$\begin{array}{ccc} R_{2} & = & \left(1+\frac{1}{\alpha }\right)\underset{\theta \in \Theta }{\sup}\int \left|q_{n}^{\alpha }\left(\theta \right)\left(x\right)-q_{\theta }^{*\alpha }\left(x\right)\right|dP_{n}\left(x\right)\\ & \leq & \underset{\theta \in \Theta }{\sup}\left(1+\frac{1}{\alpha }\right)\frac{1}{n}\sum \left|q_{n}^{\alpha }\left(\theta \right)\left(X_{i}\right)-q_{\theta }^{*\alpha }\left(X_{i}\right)\right| \end{array}$$

$$\leq C_{2}\times \underset{\theta \in \Theta }{\sup}\underset{x\in K}{\sup}\left|q_{n}\left(\theta \right)\left(x\right)-q_{\theta }^{*}\left(x\right)\right|$$

which tends to 0 in probability, making use of Proposition 4; the constant $C_{2}$ stems from condition (12).

Turn to $R_{3}.$ The class of functions $q_{\theta }^{*\alpha }$ indexed by θ satisfies the three following properties: (i) It is indexed by θ in Θ, a compact subset of $R^{d}$. (ii) Secondly it is continuous in θ for all *x* in *K*. (iii) Thirdly the function *F* defined on *K* by $F\left(x\right):=\sup_{\theta \in \Theta }\left|q_{\theta }^{*\alpha }\left(x\right)\right|$ is such that

$$\int F\left(x\right)dP_{0}\left(x\right)<\infty .$$

Whenever these three facts hold, then

$$R_{3}=\left(1+\frac{1}{\alpha }\right)\underset{\theta \in \Theta }{\sup}\left|\int q_{\theta }^{*\alpha }\left(x\right)d\left(P_{n}-P_{0}\right)\right|$$

tends to 0 in probability since ${\left\{q_{\theta }^{*\alpha }\right\}}_{\theta }$ is a Glivenko–Cantelli class of functions, making use of [28], Chapter 1.6.

### Appendix A.9. Proof of Lemma 6

Denote $q_{\theta_{0}}^{*}$ the projection of $P_{0}$ on $M_{E}$; thus, $\theta_{0}:=\arg\inf_{\theta \in \Theta }R_{\alpha }\left(Q_{\theta }^{*},P_{0}\right)$. For any θ∈Θ, let $Q_{\theta }^{*}$ be the projection of $P_{0}$ on $M_{\theta_{E}}$; hence,

$$R_{\alpha }\left(Q_{\theta }^{*},p_{0}\right)\geq R_{\alpha }\left(Q_{\theta_{0}}^{*},P_{0}\right).$$

We prove that equality cannot hold in the above display. Let $|\theta -\theta_{0}|>\epsilon$. Assume that there exists some $\theta_{1}$ with

$$d(q_{\theta_{1}}^{*},q_{\theta_{0}}^{*})>\delta$$

such that

(A6) $$R_{\alpha }\left(Q_{\theta_{1}}^{*},P_{0}\right)=R_{\alpha }\left(Q_{\theta_{0}}^{*},P_{0}\right),$$

which cannot hold, since $\theta_{0}^{*}$ achieves the minimum of $R_{\alpha }\left(Q_{\theta }^{*},P_{0}\right)$ on θ, and $Q⟶R_{\alpha }\left(Q,P_{0}\right)$ is strictly convex.

### Appendix A.10. Proof of Lemma 7

By definition $M_{n}\left(\theta \right)<R_{\alpha }\left(Q_{\theta }^{*},P_{n}\right)$.

Since $R_{\alpha }\left(Q_{\theta }^{*},P_{n}\right)-R_{\alpha }\left(Q_{\theta }^{*},P_{0}\right)\overset{P}{⟶}0$ by the Glivenko–Cantelli Theorem, it follows that

$$M_{n}\left(\theta \right)\leq R_{\alpha }\left(Q_{\theta }^{*},P_{0}\right)+\eta_{n}$$

for *n* large enough, where $\eta_{n}\overset{P}{⟶}0.$
